# Imaging, clinical, and histopathological challenges in diagnosing retroperitoneal extraskeletal osteosarcoma

**DOI:** 10.3389/fonc.2025.1454055

**Published:** 2025-02-26

**Authors:** Jiro Ichikawa, Tomonori Kawasaki, Kojiro Onohara, Masanori Wako, Naofumi Taniguchi, Satoshi Ochiai, Hirotaka Haro

**Affiliations:** ^1^ Department of Orthopaedic Surgery, Interdisciplinary Graduate School of Medicine, University of Yamanashi, Chuo, Japan; ^2^ Department of Pathology, Saitama Medical Center, Saitama Medical University, Kawagoe, Japan; ^3^ Department of Radiology, Interdisciplinary Graduate School of Medicine, University of Yamanashi, Chuo, Japan; ^4^ Department of Orthopaedic Surgery, National Hospital Organization (NHO) Kofu National Hospital, Kofu, Japan

**Keywords:** extraskeletal osteosarcoma, dedifferentiated liposarcoma, retroperitoneal, imaging, histopathology, calcification

## 1 Introduction

Extraskeletal osteosarcoma (EO) is a malignant mesenchymal neoplasm characterized by the production of osteoid tissue without connection to the skeletal system (1). EO is rare and accounts for approximately 6% of all osteosarcomas. EO frequently occurs in middle-aged and older individuals in the lower extremities and retroperitoneum (1). Recently, Sapiano et al. reported a case of primary retroperitoneal EO with extensive calcification (2). Although they differentiated retroperitoneal EO from cystadenocarcinoma and gastrointestinal stromal tumors, there are other retroperitoneal tumors showing calcification, which include liposarcoma, undifferentiated pleomorphic sarcoma, rhabdomyosarcoma, and neurogenic tumors (3). Among these tumors, in our opinion, dedifferentiated liposarcoma (DDLPS), especially with osteosarcomatous elements, may involve a critical differential diagnosis, as both EO and DDLPS share not only the clinical presentation but also the imaging and pathology profile. In this paper, we aim to highlight that DDLPS and EO both have similar features, including clinical features, imaging, and histopathology. In addition, we discuss their treatment and prognosis.

## 2 Discussion

A case series revealed a median age of 58 years for patients with EO, with a male-to-female distribution of 59:41% (4). Additionally, 80% of the tumors were deep tumors, with a median maximal diameter of 8.5 cm; these tumors most frequently affected the thigh (4). DDLPS is a subtype of liposarcoma, with a median patient age of 64 years and a male-to-female sex distribution of 65:35% (5). The tumor size is greater than 10 cm in 60% of cases, most frequently affecting the retroperitoneum, which is correlated not only with shorter overall survival (OS) but also with dedifferentiation from atypical lipomatous tumor/well-differentiated liposarcoma (ALT/WDLPS) (5, 6). Approximately 5–10% of patients with DDLPS show heterogeneous differentiation (6), with the most common differentiation being related to myogenic and osteosarcomatous/chondrosarcomatous elements (6). The clinical features of EO and DDLPS may be similar.

Regarding the imaging features, although DDLPS is termed as a liposarcoma, approximately 70% of cases show no fat component (7) suggesting that even with retroperitoneal occurrence, it is almost impossible to suspect DDLPS without an ALT/WDLPS component. On the other hand, the MRI features of EO are characterized as necrotic changes in 97%, hemorrhagic changes in 38%, and heterogenous T2 (varying degrees) in 100% of cases (8); surprisingly, the corresponding percentages for DDLPS are 72%, 41%, and 95%, respectively (7), suggesting a great overlap of these features. Conversely, calcification was seen in only 60% of cases with EO, and the volume of calcification was less than 10% of the whole tumor in approximately 70% of cases, suggesting that massive calcification may be rare (8). In EO, the presence of calcification is associated with a worse prognosis (8); however, in cases of retroperitoneal DDLPS, the presence of calcification was correlated not with OS but with local recurrence-free survival (LRFS). Interestingly, a retroperitoneal location in DDLPS with calcification was related to LRFS only, and not to OS or distant metastasis-free survival (9).

Next, concerning the histopathology, DDLPS and ALT/WDLPS both share the characteristics of high amplification in the 12q14-15 chromosomal region, including MDM2 and CDK4, which represents a great advantage for establishing the correct diagnosis using immunohistochemistry (IHC) and fluorescence *in-situ* hybridization (FISH) (10, 11). The sensitivity of MDM2 and CDK4 amplification in IHC was reported as 100% and 83%, respectively (11). On the other hand, the specificity of MDM2 and CDK4 was relatively low, as some sarcomas, including myxofibrosarcoma and undifferentiated pleomorphic sarcoma, also show positivity (11). The utility of p16 in combination with MDM2 and CDK4 was proposed to increase specificity (11). Interestingly, 30–40% of EO cases were positive for both MDM2 and CDK4 across staining methods. MDM2 FISH is useful in DDLPS, and its sensitivity is more than 90% (8); however, MDM2 amplification is also observed in more than 30% of EO cases (12). Although Special AT-rich binding protein-2, an osteoblastic marker, is helpful in the diagnosis of EO (1), it is currently unknown how useful these markers are in differentiating DDLPS, especially DDLPS with osteosarcoma-like elements. In addition, calcification may also be observed in malignant peripheral nerve sheath tumors, and deletion of H3K27me3 is a useful marker. Unfortunately, deletion of H3K27me3 can also be observed in EO, although to a lesser extent than that of MDM2 (12). Considering the overlap of these markers between EO and other sarcomas, it can be speculated that EO is a heterogeneous disease containing several subsets with distinct clinicopathological and molecular features. To overcome such diagnostic difficulties and to elucidate the nature of the disease in EO from the perspective of molecular pathological features, genomic and transcriptomic analyses using RNA sequencing and next-generation sequencers are being conducted in osteosarcoma, which is considered to be essentially the same as EO (13, 14). By extracting RNA from osteosarcoma and simultaneously collecting samples from normal bone, it is possible to identify markers that are more specific to osteosarcoma by comparing the gene expression of both. It is necessary to analyze both significantly increased and decreased gene expressions. Furthermore, pathways related to these genes can also be elucidated (15). Identifying such markers and related signaling pathways contributes to more accurate diagnosis and potentially, to new therapeutic targets. In this analysis, reference samples are extremely important, and their quality must also be ensured (16). Genomics can provide very precise information and help differentiate these cancers (17). If such analyses progress in EO as well as osteosarcoma and novel and new target molecule will be discovered, it may clarify not only the diagnosis of EO but also the similarities or differences between EO and osteosarcoma.

Finally, the prognosis of EO still remains poor, with a reported 5-year OS rate of 37–52% (1). The main treatment strategy is surgery, and R0 surgery is associated with better OS and lower recurrence (4). On the other hand, tumor depth and maximal diameter, which have been reported as prognostic factors (4), may be involved in retroperitoneal tumors. Considering these situations, the development of adjuvant therapy in EO is urgent; unfortunately, the efficacy of chemotherapy in EO has been limited, suggesting routine chemotherapy is not recommended for patients with localized EO (18). On the other hand, regarding the differences in chemotherapy regimens, the 5-year disease-free survival when using osteosarcoma-type chemotherapy was 56.3%, which is higher than the 45.2% of soft tissue sarcoma-type chemotherapy (19). The efficacy for inoperable and metastatic cases, such as the one reported by Sapiano et al. (2), also remains unclear. Using radiotherapy for EO affects local recurrence but not OS (4). Considering the resistance to radiotherapy observed in conventional osteosarcomas, EO might show a behavior similar to that of soft tissue sarcoma (4). Regarding chemotherapy for DDLPS, the use of doxorubicin, either as a single agent or in combination with ifosfamide, has been reported. Although the combination of doxorubicin and ifosfamide was expected to have a clinical effect, it exhibited no significant improvement in OS; moreover, side effects, such as hematologic toxicity, were frequently observed (20). To overcome the limited efficacy, recently, clinical trials for MDM2- and CDK4-targeting drugs have been conducted (20). If favorable results are obtained, they are expected to be used for treating MDM2- or CDK4-positive EO. The prognosis of both EO and DDLPS is poor, and it is well known that a retroperitoneal origin often makes complete resection difficult due to the presence of vital organs, such as the kidney and intestinal tract. In this aspect, it is crucial for physicians to know whether the efficacy of adjuvant therapy, including chemotherapy and radiation, will remain clear.

In conclusion, this article focused on DDLPS, which is very difficult to differentiate from EO from the clinical, imaging, and pathological perspectives. Although EO is presumed to be a “heterogeneous” condition, the analysis of further cases, especially by genomics, may reveal details of its nature and correlations with other types of sarcoma.

## References

[1] Yamashita K Hameed M. Extraskeletal osteosarcoma. In: WHO classification of tumours editorial board. WHO classification of tumours soft tissue and bone tumours. IARC Press, Lyon (2020). p. 224–5.

[2] Sapiano A Caruana C Spiteri N Gatt AS Degiorgio S Samuel A. Extraskeletal osteosarcoma of primary retroperitoneal origin. Lancet Oncol. (2023) 24:e284. doi: 10.1016/s1470-2045(23)00155-9 37269859

[3] Sangster GP Migliaro M Heldmann MG Bhargava P Hamidian Jahromi A Thomas-Ogunniyi J. The gamut of primary retroperitoneal masses: multimodality evaluation with pathologic correlation. Abdom Radiol (NY). (2016) 41:1411–30. doi: 10.1007/s00261-016-0735-6 27271217

[4] Heng M Gupta A Chung PW Healey JH Vaynrub M Rose PS. The role of chemotherapy and radiotherapy in localized extraskeletal osteosarcoma. Eur J Cancer. (2020) 125:130–41. doi: 10.1016/j.ejca.2019.07.029 PMC7261507 31806415

[5] Gootee J Aurit S Curtin C Silberstein P. Primary anatomical site, adjuvant therapy, and other prognostic variables for dedifferentiated liposarcoma. J Cancer Res Clin Oncol. (2019) 145:181–92. doi: 10.1007/s00432-018-2777-3 PMC11810416 30361927

[6] Dei Tos AP Marino-Enriquez A Pedeutour F. Dedifferentiated liposarcoma. In: WHO classification of tumours editorial board. WHO classification of tumours soft tissue and bone tumours. IARC Press, Lyon (2020). p. 39–41.

[7] Kawaguchi M Kato H Kobayashi K Miyazaki T Nagano A Noda Y. MRI findings to differentiate musculoskeletal dedifferentiated liposarcoma from atypical lipomatous tumor. Radiol Med. (2022) 127:1383–9. doi: 10.1007/s11547-022-01547-9 36350422

[8] Crombé A Spinnato P Righi A Leopardi MP Carpenzano M Izzo F. Imaging presentation of extraskeletal osteosarcomas on CT and MRI and correlation with patients outcome: A two-center retrospective study of 54 patients. Diagn Interv Imaging. (2023) 104:297–306. doi: 10.1016/j.diii.2023.01.009 36813659

[9] Yamashita K Kohashi K Yamada Y Ishii T Nishida Y Urakawa H. Osteogenic differentiation in dedifferentiated liposarcoma: a study of 36 cases in comparison to the cases without ossification. Histopathology. (2018) 72:729–38. doi: 10.1111/his.13421 29076540

[10] Thway K. Well-differentiated liposarcoma and dedifferentiated liposarcoma: An updated review. Semin Diagn Pathol. (2019) 36:112–21. doi: 10.1053/j.semdp.2019.02.006 30852045

[11] Kammerer-Jacquet SF Thierry S Cabillic F Lannes M Burtin F Henno S. Differential diagnosis of atypical lipomatous tumor/well-differentiated liposarcoma and dedifferentiated liposarcoma: utility of p16 in combination with MDM2 and CDK4 immunohistochemistry. Hum Pathol. (2017) 59:34–40. doi: 10.1016/j.humpath.2016.08.009 27597521

[12] Makise N Sekimizu M Kubo T Wakai S Watanabe SI Kato T. Extraskeletal osteosarcoma: MDM2 and H3K27me3 analysis of 19 cases suggest disease heterogeneity. Histopathology. (2018) 73:147–56. doi: 10.1111/his.13506 29489027

[13] Reimann E Kõks S Ho XD Maasalu K Märtson A. Whole exome sequencing of a single osteosarcoma case–integrative analysis with whole transcriptome RNA-seq data. Hum Genomics. (2014) 8:20. doi: 10.1186/s40246-014-0020-0 25496518 PMC4272536

[14] Ho XD Nguyen HG Trinh LH Reimann E Prans E Kõks G. Analysis of the expression of repetitive DNA elements in osteosarcoma. Front Genet. (2017) 8:193. doi: 10.3389/fgene.2017.00193 29250102 PMC5714928

[15] Märtson TA Kõks S Reimann E Prans E Erm T and Maasalu K. Transcriptome analysis of osteosarcoma identifies suppression of wnt pathway and up-regulation of adiponectin as potential biomarker. Genomics Discovery. (2013) 1:3. doi: 10.7243/2052-7993-1-3

[16] Fang LT Zhu B Zhao Y Chen W Yang Z Kerrigan L. Establishing community reference samples, data and call sets for benchmarking cancer mutation detection using whole-genome sequencing. Nat Biotechnol. (2021) 39:1151–60. doi: 10.1038/s41587-021-00993-6 PMC8532138 34504347

[17] Poudel BH Koks S. The whole transcriptome analysis using FFPE and fresh tissue samples identifies the molecular fingerprint of osteosarcoma. Exp Biol Med (Maywood). (2024) 249:10161. doi: 10.3389/ebm.2024.10161 38966281 PMC11222325

[18] Tsukamoto S Mavrogenis AF Angelelli L Righi A Filardo G Kido A. The effect of adjuvant chemotherapy on localized extraskeletal osteosarcoma: A systematic review. Cancers (Basel). (2022) 14:2559. doi: 10.3390/cancers14102559 35626164 PMC9139294

[19] Campos F Téres R Sebio A Bettim BB Martinez-Trufero J. Survival differences of patients with resected extraskeletal osteosarcoma receiving two different (Neo)Adjuvant chemotherapy regimens: A systematic review and meta-analysis. Clin Oncol (R Coll Radiol). (2023) 35:e720–e7. doi: 10.1016/j.clon.2023.09.009 37777356

[20] Gahvari Z Parkes A. Dedifferentiated liposarcoma: systemic therapy options. Curr Treat Options Oncol. (2020) 21:15. doi: 10.1007/s11864-020-0705-7 32026050

